# A Comparative Study of Ganglion Cell Complex Thickness Changes in Diabetic Macular Edema and Central Retinal Vein Occlusion Macular Edema: An Optical Coherence Tomography Study

**DOI:** 10.7759/cureus.30609

**Published:** 2022-10-23

**Authors:** Swapneel Mathurkar, Sachin Daigavane, Chrisann Saldanha

**Affiliations:** 1 Department of Ophthalmology, Jawaharlal Nehru Medical College, Datta Meghe Institute of Medical Sciences University, Wardha, IND; 2 Department Of Ophthalmology, Jawaharlal Nehru Medical College, Datta Meghe Institute of Medical Sciences University, Sawangi, Wardha, IND

**Keywords:** ganglion cell complex, diabetic retinopathy, oct (optical coherence tomography), central retinal vein occlusion (crvo), diabetic macular oedema

## Abstract

Aim

This study aims to compare the ganglion cell complex changes in diabetic macular edema (DME) and central retinal vein occlusion (CRVO) macular edema using optical coherence tomography (OCT).

Methods

This was a hospital-based cross-sectional study conducted for six months. All patients having DME and CRVO macular edema presenting to the Ophthalmology Department at Acharya Vinobha Bhave Rural Hospital were included in the study. A detailed and comprehensive ophthalmic examination was performed, and OCT was done for each patient.

Results

The incidence of both DME and CRVO macular edema were both found to be maximum in the age group of 61-69 years. DME is more common in males (62.86%) than females (37.14%); the same was observed in CRVO group: 54.29% were males and 45.71% were females. Macular edema showed a mean value of 370.11 in DME and 428.71 in CRVO. Thus, the CRVO group showed more macular edema than the DME group. The ganglion cell complex thickness showed a mean value of 58.47 in DME and 66.77 in the CRVO group, implying that the thickness reduced significantly in the DME group.

Conclusion

OCT provides quantitative measurement of the ganglion cell complex thickness, which helps monitor the course of macular edema secondary to CRVO and diabetes Mellitus and thereby provides an assessment of the prognosis of the disease as these two diseases in particular are major causes of blindness worldwide, and timely care and management can help in altering its course.

## Introduction

Diabetes mellitus is an endemic disease and has a rapidly increasing prevalence worldwide [1]. Diabetic retinopathy is an important ocular manifestation of diabetes mellitus. More than 50% of patients with insulin-dependent diabetes mellitus develop retinopathy in around 15 years [1]. In noninsulin-dependent diabetes, the risk of retinopathy increases with the duration of the disease; however, it is generally accompanied by a history of hypertension and smoking [2]. Pathogenesis of this disease consists of micro- and macrovascular complications. The microvascular complications are mainly microaneurysms and retinal microhemorrhages. Macular edema occurs in the majority of eyes and is a significant cause of the diminution of vision in diabetic retinopathy [2].

Central retinal vein occlusion (CRVO) is an ocular pathology that significantly impacts the vision as the retina's veins get engorged with blood and become tortuous [2]. The retina is filled with many hemorrhages, and many tortuous vessels start to develop around the optic disc. Eventually, in some types of CRVO, the affected retina becomes atrophic with the pigmentary changes. Macular edema is an important consequence of CRVO, which causes significant visual impairment. The main reason is the breakdown of the blood-retinal barrier with the accumulation of fluid in the intra-retinal layer in the macula, causing an increase in vascular endothelial growth factor in the vitreous cavity, thereby increasing macular edema [3].

Optical coherence tomography (OCT) [4] is an effective diagnostic tool; it helps perform cross-sectional images of biological tissues within less than 10-micron axial resolution using light waves. OCT is a crucial tool in retinal disorders as it allows quantitative measurements of retinal thickness. The ganglion cell complex comprises three layers of the retina, namely the retinal nerve fiber layer, the ganglion cell layer, and the inner plexiform layer [5]. Both diabetic macular edema (DME) and CRVO macular edema have repercussions on the thickness of this layer, thereby making OCT an essential tool as it helps in measuring the thickness of this layer [6,7]. OCT helps quantify the structural changes of different cellular layers of the retina, which thereby helps in identifying potential markers for the disease and monitoring the progression of the disease [8]. Not many studies have been conducted about this in this region.

Objectives

This study aims to compare the ganglion cell complex changes in DME and CRVO macular edema using OCT. We aim to evaluate the thickness of the ganglion cell complex layer in people with DME using OCT and to evaluate the thickness of the ganglion cell complex layer in CRVO patients with macular edema using OCT and to compare the changes between the ganglion cell complex (GCC) layer in DME and CRVO patients with macular edema.

## Materials and methods

This is a hospital-based cross-sectional study conducted at a rural hospital in Central India for six months, from December 2020 to May 2021. All patients having DME and CRVO macular edema were included in the study.

Inclusion criteria

Patients above 18 years of age, patients having insulin and noninsulin-dependent diabetes mellitus with non-proliferative and proliferative diabetic retinopathy with macular edema, and patients having either ischemic or non-ischemic CRVO with macular edema were included in the study

Exclusion criteria

Patients below 18 years of age, patients with gross retinal pathologies such as degenerative myopia, retinitis pigmentosa, and retinal and choroidal dystrophies and degenerations, or any other retinal disorders affecting the ganglion cell complex and retinal nerve fiber layer, patients with glaucoma or those with intraocular pressure > 21 mmHg in either eye, patients who underwent refractive surgery, patients unable or unwilling to provide informed consent, and patients who have received anti-VEGF treatment for DME- and CRVO-associated macular edema were excluded.

Sampling procedure

A total of 70 patients (70 eyes; (35 DME and 35 CRVO macular edema) who presented to the hospital during the study period were included and distributed on the basis of fundus examination and OCT examination performed by an experienced vitreoretinal surgeon (based on a 95% confidence interval, the prevalence is 0.8% in India and the desired margin of error of 5%=0.05) [9].

Statistical analysis was conducted using descriptive and inferential statistics with the chi-square test. Student’s unpaired t-test and SPSS Version 27.0 (IBM Corp., Armonk, NY) were used in the analysis, and p<0.05 was considered the level of significance.

## Results

This study included 70 patients (70 eyes) divided into two groups of 35 each depending on whether they suffered from macular edema due to CRVO or DME. Group 1, included patients having macular edema due to CRVO and group 2 included patients having macular edema due to diabetic retinopathy, both proliferative and non-proliferative.

**Figure 1:**
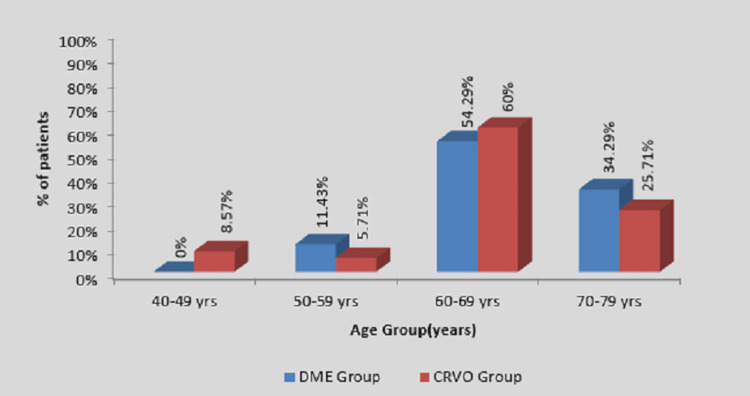
Distribution of patients according to age in years in two groups DME, diabetic macular edema; CRVO, central retinal vein occlusion

Table 1 and Figure 1 show the age-wise distribution between the two groups, where maximum patients were found in the age group of 60-69 years in both groups, with a mean age of 63.97 ± 7.62 in the CRVO group (60%) and 66.34 ± 6.05 in the DME group (54.29%).

**Table 1:** Distribution of patients according to their age in years in two groups SD, standard deviation; NS, not significant

Age Group (years)	Diabetic Macular Edema Group	Central Retinal Vein Occlusion Group	Total	Χ^2^ value
40-49	0 (0%)	3 (8.57%)	3(4.29%)	4.19; p=0.11, NS
50-59	4 (11.43%)	2 (5.71%)	6(8.57%)	
60-69	19 (54.29%)	21 (60%)	40(57.14%)	
70-79	12 (34.29%)	9 (25.71%)	21(30%)	
Total	35 (100%)	35 (100%)	70(100%)	
Mean ± SD	66.34 ± 6.05	63.97 ± 7.62	65.15±6.93	
Range	50-76 years	40-75 years	40-76 years	

**Figure 2:**
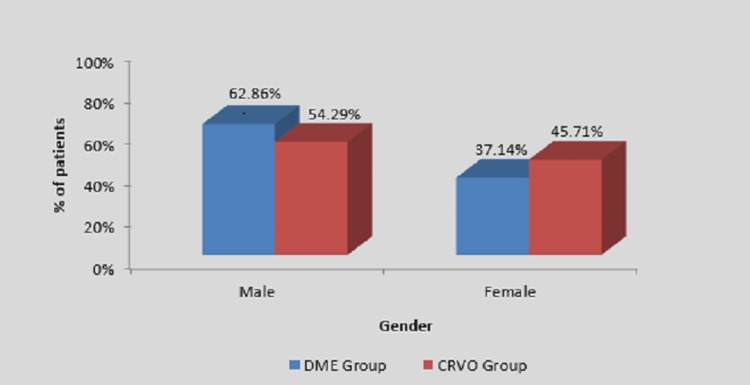
Distribution of patients according to gender in two groups DME, diabetic macular edema; CRVO, central retinal vein occlusion

As shown in Table 2 and Figure 2, the number of males in both groups was more than in females.

**Table 2:** Distribution of patients according to gender in two groups NS, not significant

Gender	Diabetic Macular Edema Group	Central Retinal Vein Occlusion Group	Total	Χ^2^ value
Male	22 (62.86%)	19 (54.29%)	41(58.57%)	0.53; p=0.46, NS
Female	13 (37.14%)	16 (45.71%)	29(41.43%)	
Total	35 (100%)	35 (100%)	70(100%)	

**Figure 3:**
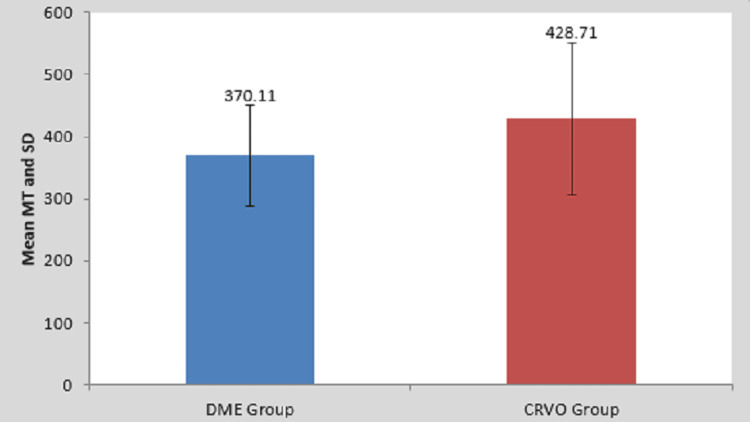
Comparison of macular edema thickness among the patients of two groups MT, macular thickness; SD, standard deviation

Among both groups, it is shown that in the DME group, macular edema thickness showed a mean of 370.11, and in the CRVO group, macular edema showed a mean of 428.71 (Table 3 and Figure 3). Thus, macular edema thickness in this study was shown to be more in the CRVO group than in the DME group.

**Table 3:** Comparison of macular edema thickness among the patients of two groups

Group	Normal	Mean	Standard Deviation	Standard Error Mean	t-Value
Diabetic macular edema group	35	370.11	81.45	13.76	2.36; p=0.021, significant
Central retinal vein occlusion group	35	428.71	121.90	20.60	

**Figure 4:**
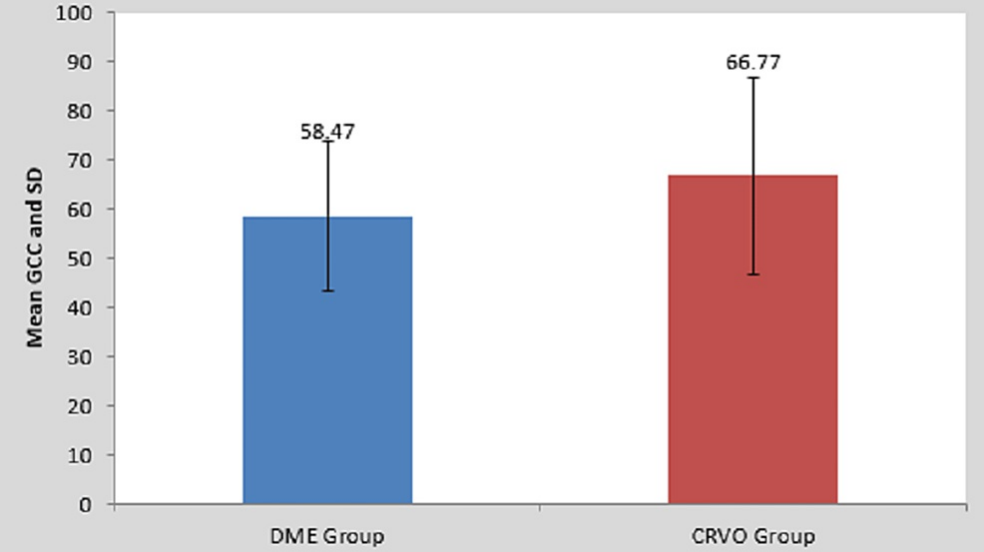
Comparison of ganglion cell complex among the patients of two groups

In both groups, the ganglion cell complex thickness was 58.47 in the DME group and 66.77 in the CRVO group. Thus this showed that ganglion cell thickness was reduced to a greater extent in patients with DME than with CRVO (Table 4 and Figure 4).

**Table 4:** Comparison of ganglion cell complex among the patients of two groups

Group	Normal	Mean	Standard Deviation	Standard Error Mean	t-Value
Diabetic macular edema group	35	58.47	15.24	2.57	2.02; p=0.046, significant
Central retinal vein occlusion group	35	66.77	19.90	3.36	

## Discussion

This is a hospital-based cross-sectional study that analyzes the changes in the ganglion cell complex thickness in patients with DME and CRVO macular edema. In our study, a total of 70 patients (70 eyes) were included, with 35 patients each in the CRVO macular edema group and the DME group. The incidence of CRVO macular edema and DME is somewhat similar in both groups, 60-69 years of age, with a mean age of 66.97 in the former and 63.94 in the latter. The incidence of CRVO macular edema and DME is more in males than females in this study. The ganglion cell complex changes in both groups showed thinning of GCC complex in both groups, slightly more in the DME group (58.47) than in the CRVO macular edema group (66.77). Macular edema in both groups was checked, and the DME group showed a mean of 370.11 and the CRVO group showed a mean of 428.71.

Neurovascular coupling is a mechanism that affects the retinal blood flow, and its effects differ in patients with diabetes mellitus and those with CRVO [9]. In diabetics, hyperglycemia activates pathways such as polyol and hexosamine, producing free radicals and advanced glycation products [2,10]. These pathways are responsible for the damage to the neural retina as they cause inflammation and ischemia, which, in turn, cause neural degeneration [11]. The most significant feature of diabetic retinopathy is neural apoptosis, and some studies have suggested the involvement of retinal ganglion and amacrine cells [2,10]. It was coupled with reactive gliosis and is responsible for the decrease in ganglion cell complex thickness in diabetic retinopathy, which can be studied effectively with OCT. Studies by van Dijk et al. showed a reduction in ganglion cell complex thickness in patients with but not without diabetic retinopathy [12,13]. Ng et al. reported ganglion cell complex loss in patients with and without diabetic retinopathy; the loss was progressive in advanced diabetic retinopathy, with a decrease in inner retinal layer thickness, as was also shown in our study [7,14].

In cases with CRVOs, damage to the photoreceptors and permanent neuronal damage due to hypoxia of the retina are causes of permanent visual loss. Fluid accumulation within the layers of the retina is responsible for Muller cell ballooning and retinal degeneration. Over a period of time, as the macular edema continues to progress, it can cause irreversible damage to neurons. It is one of the crucial causes of damage to the ganglion cell complex, leading to decreased thickness [15,16]. Secondly, toxic effects of anti-VEGF on ganglion cells could occur. Similar results were reported in this study and were seen on OCT.

However, this study also has its limitations. Firstly, the sample size for this study is small. More extensive studies with a greater sample size must confirm these results. We could not take long-term follow-up in these patients, and thus the ganglion cell complex thickness could not be analyzed over time. Also, the groups studied were not age- and gender-matched, which could further lead to a discrepancy in the results.

## Conclusions

DME and CRVO macular edema are essential factors causing visual impairment. It is crucial to analyze this in the further management of these conditions and visual prognosis in both largely depends on the ganglion cell complex thickness which is measured on OCT. OCT provides quantitative measurement of the ganglion cell complex thickness in macular edema, which helps monitor the course of macular edema secondary to the above diseases and thereby provides assessment of the prognosis of the disease.
